# Impact of breast size on dosimetry and radiobiology of VMAT left‐sided breast‐conserving conventional fractionation radiotherapy under setup errors

**DOI:** 10.1002/acm2.70151

**Published:** 2025-07-13

**Authors:** Chao Zheng, Danting Cai, Qingsong Zhong, Wen Dou, Binbin Yuan

**Affiliations:** ^1^ Department of Radiology Zhujiang Hospital Southern Medical University Guangzhou Guangdong People's Republic of China

**Keywords:** breast, radiotherapy, setup errors, treatment planning, VMAT

## Abstract

**Purpose:**

The aim of this retrospective study was to investigate the impact of setup errors on the dosimetry and radiobiology of left‐sided breast cancer (BC) patients with different breast sizes undergoing conventionally fractionated volumetric modulated arc therapy (VMAT) radiotherapy.

**Methods:**

A total of 36 BC patients who underwent breast‐conserving surgery were enrolled in the study. Setup errors were simulated through isocenter shifts in six directions. Differences in dosimetric and radiobiological parameter variations between the large breast group (volume > 975 cm^3^) and the small breast group were compared under the same setup errors, relative to the original plan for the planning target volume (PTV) and organs at risk (OARs).

**Results:**

When the isocenter error reached 2.5 mm, notable dosimetric deviations were observed, except for the PTV in the right direction and the heart in the superior direction, which remained stable. At a 5 mm isocenter error, the small breast group demonstrated greater dosimetric variability in the PTV compared to the large breast group. However, the heart showed less variation in the small breast group. Additionally, for both 2.5 and 5 mm isocenter errors, the small breast group had smaller changes in the NTCP for the heart than the large breast group.

**Conclusions:**

In conventionally fractionated VMAT radiotherapy for left‐sided breast cancer, isocenter setup errors affect the dosimetric and radiobiological outcomes of the PTV and OARs to varying degrees, depending on setup errors and breast size. When the setup error exceeds 2.5 mm, patients with larger breasts experience more pronounced increases in heart dose and Normal Tissue Complication Probability (NTCP) than those with smaller breasts, particularly in the right and posterior directions. Therefore, stricter setup error thresholds (e.g., ≤2.5 mm) and more frequent imaging guidance (e.g., ≥2 cone‐beam computed tomography (CBCT) verifications per week) are recommended for this patient group.

## 1 BACKGROUND

Breast cancer (BC) has surpassed lung cancer as the most common cancer globally, with 2.3 million new cases diagnosed worldwide in 2020.^1^ In China, BC ranks as the leading cause of malignant tumors among women in terms of incidence and is the sixth leading cause of cancer‐related mortality. With advances in breast surgery, approximately 60% of patients with early‐stage BC now opt for breast‐conserving surgery. This procedure effectively removes the tumor while preserving the breast, leading to a notable improvement in patients' postoperative quality of life.^2^, ^3^ Adjuvant radiotherapy is strongly recommended for patients with early‐stage BC following breast‐conserving surgery, as it significantly reduces the risk of local recurrence and distant metastasis compared to surgery alone, while achieving survival rates comparable to those of radical mastectomy.^4^, ^5^, ^6^, ^7^ Volumetric Modulated Arc Therapy (VMAT) represents one of the most advanced radiotherapy techniques available. By dynamically adjusting the gantry rotation speed, beam dose rate, and the position of the multi‐leaf collimator, VMAT optimizes dose intensity distribution across the target. This not only enhances dose uniformity within the target area but also minimizes radiation exposure to organs at risk (OARs), such as the heart and lungs, making it a preferred approach in BC adjuvant radiotherapy.^8^ Moreover, compared to other intensity‐modulated radiotherapy techniques, VMAT substantially decreases the number of monitor units (MUs) required and shortens treatment delivery times. These improvements help to lower the dose uncertainties associated with intra‐fraction patient movement, further enhancing treatment precision and safety.^9^

Inter‐fraction error is a critical factor affecting the accuracy of radiation dose delivery, primarily stemming from equipment uncertainties and the positioning techniques used by radiation therapists.^10^ When setup errors occur, the steep dose‐response curves generated by VMAT are substantially altered, impacting patient outcomes in terms of local tumor control and normal tissue complications.^11^, ^12^, ^13^, ^14^ Liao et al. reported that a 3 mm isocenter shift in the right, inferior, and posterior directions led to significant dosimetric deviations in postoperative breast cancer patients, with the heart and lungs experiencing the most pronounced impacts.^11^ Similarly, Johnson et al. investigated dose variations in non‐small cell lung cancer patients with thoracic tumors due to setup errors. Their findings demonstrated a significant correlation between these errors and overall patient survival rates.^13^ However, most existing studies on the impact of setup errors mainly compare radiotherapy techniques (e.g., VMAT vs. intensity‐modulated radiation therapy (IMRT)) or image‐guidance methods (e.g., Cone‐beam computed tomography (CBCT) vs. electronic portal imaging device (EPID)).^15^, ^16^, ^17^, ^18^ They generally overlook patient anatomical heterogeneity, especially the role of breast volume in error sensitivity. For example, Jensen et al. systematically compared the dosimetric stability of VMAT and three‐dimensional conformal radiation therapy (3D‐CRT) in locally advanced breast cancer radiotherapy under deep inspiration breath‐hold (DIBH) conditions in the presence of setup errors.^18^ Similarly, Mukundan et al. demonstrated that EPID technology can help quantify and reduce setup errors in breast radiotherapy, subsequently affecting dose variations in the target and organs at risk.^16^ However, these studies did not account for anatomical features, leaving a critical gap in understanding the relationship between breast size and setup error sensitivity.

It is well established that breast size is closely associated with target coverage and the dose delivered to adjacent OARs in clinical radiotherapy.^19^, ^20^, ^21^ A recent retrospective clinical study involving 4688 BC patients treated with Whole Breast Radiation Therapy (WBRT) revealed that patients with larger breasts exhibit a notably higher mean heart dose (MHD). Specifically, the study found that under a hypofractionation strategy, each 1 cm increase in breast thickness results in a 1.7% increase in MHD.^21^ Another study reported that patients with breast volumes exceeding 1000 cm^3^ receive higher doses to the ipsilateral lung compared to those with smaller breasts.^19^ Such volume‐dependent dosimetric deviations may be further amplified by setup errors.

This study pioneers a systematic investigation of dosimetric and radiobiological responses to setup errors in left‐sided breast cancer patients with varying breast volumes under conventionally fractionated VMAT. Additionally, it elucidates the combined impact of breast volume and error direction on both the target and OARs.

## 2 MATERIALS AND METHODS

### 2.1 Patients and volumes delineation

Thirty‐six patients diagnosed with left‐sided BC and treated with breast‐conserving surgery were randomly selected for this retrospective study. The median patient age was 52 years (range: 27–72 years), and the average body mass index (BMI) was 23.54 (range: 19–35.5). All patients underwent adjuvant radiotherapy using VMAT in our department in 2023. All participants received radiotherapy for the first time, with no interruptions or early terminations. In accordance with protocols established by previous investigators, a planning target volume (PTV) exceeding 975 cm^3^ was defined as indicative of a large breast.^22^, ^23^, ^24^ Accordingly, 18 patients were assigned to the large breast group, while the remaining patients were placed in the small breast group. Written informed consent was obtained from all participants, and the data were used exclusively for research purposes.

All patients were fully conscious and able to follow the physician's instructions. Patients were positioned in the supine position using a breast board with arm supports, which allowed free breathing and fully exposed the breast and chest wall. Imaging was performed using a Philips computed tomography scanner (Discovery CT590 RT, GE, Boston, USA), which included both enhanced Computed Tomography (CT) and four‐dimensional computed tomography (4D‐CT) scans, with a slice thickness of 2.5 mm. The scanning range extended from the superior margin of the cricoid cartilage to 2 cm below the diaphragm. The acquired images were transferred to an intelligent radiotherapy contouring system (Pvmed v1.0.0, Pvmed, Guangzhou, China) for OAR contouring. An experienced radiation oncologist delineated the clinical target volume (CTV) in accordance with ICRU Report 83 guidelines. The PTV was then generated by expanding the CTV outward by 5 mm, while ensuring it remained at least 2 mm from the skin surface.

### 2.2 Plan design

All treatment plans were created using the Monaco treatment planning system (Monaco v6.1, Elekta AB, Stockholm, Sweden) and prescribed a total dose of 50 Gy to be delivered in 25 fractions. Patients received treatment on an Elekta Axesse linear accelerator using 6 MV photon beams. The setup employed two continuous VMAT fields—one rotating clockwise and the other counterclockwise—each covering approximately 240°, with control points spaced at 2° intervals.

Before each treatment fraction, the radiation therapist positioned the patient according to the initial setup, aligning the skin marks with room lasers. CBCT images were then acquired using the X‐ray Volumetric Imaging (XVI) system. CBCT imaging was performed weekly, with a total of five scans per patient throughout the treatment course. The initial displacement between CBCT and planning CT images was determined through bony registration. Subsequently, the radiation therapist made precise adjustments until the target and anatomical structures were optimally aligned in the transverse, sagittal, and coronal planes. A setup error of less than 5 mm in all directions was deemed acceptable. If any setup error exceeded this threshold, the images were re‐acquired, and the setup was recalculated. The magnitude and direction of the clinical errors were reviewed and confirmed by the physician before being recorded. Five sets of clinical errors were introduced into the treatment planning by adjusting only the isocenter position. The original gantry angle, collimator angle, and optimization parameters remained unchanged. The dose was recalculated to generate the clinical plan, resulting in five new plans per patient.

Different isocenter positions were simulated and evaluated using treatment planning software to assess inter‐fraction setup errors. Specifically, the isocenter of each plan was shifted by 2.5 mm and 5 mm in six directions: right (x−), left (x+), inferior (y−), superior (y+), posterior (z−), and anterior (z+). The corresponding perturbed plans were recalculated on the planning CT, keeping all other parameters unchanged except for the isocenter position. As a result, 12 new plans were generated for each patient.

### 2.3 Dosimetry and radiobiology calculations

The quality of the treatment plans was assessed using both dosimetric and radiobiological parameters. Metrics for the planning target volume (PTV) included $V_{95}$ (the volume receiving 95% of the prescribed dose), $D_{98\%}$ (the minimum dose delivered to 98% of the target), as well as $D_{95\%}$ and $D_{2\%}$. For organs at risk (OARs), the evaluated parameters were $V_{5}$, $V_{20}$, and the mean dose for the left lung; $V_{10}$ for the right breast along with its mean dose; and $V_{5}$, $V_{10}$, $V_{20}$ and mean dose for the heart.

The Normal Tissue Complication Probability (NTCP) parameter, derived from the dose distribution and radiobiological characteristics of the structure, quantified the risk of complications in OARs.^25^ The Equivalent Uniform Dose (EUD) model was employed to calculate NTCP, using the following foundational formula^26^, ^27^:

$$EUD={\left(\sum_{i=1}\left(v_{i}EQD_{i}^{a}\right)\right)}^{\frac{1}{a}}$$

Where $v_{i}$ represents the i‐th partial volume receiving a biological equivalent physical dose of $EQD_{i}$ and a is a dimensionless factor associated with OAR. The value of $EQD_{i}$ was calculated using the following formula:

$$EQD_{i}=D_{i}\;\frac{\frac{\alpha }{\beta }+D_{f}}{\frac{\alpha }{\beta }+2}$$

Where $\frac{\alpha }{\beta }$ represents the linear quadratic parameter for each irradiated tissue and $D_{f}$ denotes the dose per fraction. The NTCP value was derived using the following formula:

$$NTCP=\frac{1}{1+{\left(\frac{TD_{50}}{EUD}\right)}^{4r_{50}}}$$

Here, $TD_{50}$ represents the tolerance dose for a 50% complication rate for an irradiated organ, while $r_{50}$ denotes a dimensionless model parameter specific to each tumor type. The parameters were derived from previously published studies, as summarized in Table 1.^28^, ^29^ All calculations were carried out using Python 3.8.

**TABLE 1:** Radiobiologic parameters used for calculating the NTCP.

Structure	a	$r_{50}$	${\mathrm{TD}}_{50\;}$(Gy)	α/β(Gy)
Lung	1	2	24.5	3
Heart	3	3	50	2.5

### 2.4 Statistical analysis

All results of this study were analyzed using SPSS 19.0 software (INM Corp., Armonk, New York, U.S.), and are presented as mean ± standard deviation. The Shapiro‐Wilk test was used to assess the normality of the data. A paired‐samples t‐test was performed for data following a normal distribution; Otherwise, the Wilcoxon signed‐rank test was applied. Statistical significance was defined as p < 0.05.

## 3 RESULT

### 3.1 Effect of setup errors on dosimetry

Tables 2 and 3 summarize the mean ± standard deviation of dosimetric parameters for the target and OARs in the new plans generated after isocenter shifts of 2.5 mm or 5 mm in six directions. For a 2.5 mm shift in any direction, the differences in $V_{95\%},D_{98\%}$, $D_{95\%}$ and $D_{2\%}$ for the PTV between the shifted and original plans were statistically evident, except for $D_{2\%}$ in the right direction. However, all changes remained within clinically acceptable limits. Notably, in the left, superior, and anterior directions, the PTV doses—except $D_{2\%}$—were lower than in the original plan, with the magnitude of differences increasing as setup errors grew larger. At a 5 mm shift, the maximum mean differences in $V_{95\%},D_{98\%}$, $D_{95\%}$ and $D_{2\%}$ for the PTV between the shifted and original plans were 6.12%, 757.17cGy, 497.67cGy, and 43.45cGy, respectively. The first three occurred in the anterior direction, while the last was observed in the posterior direction. For OARs, except for the heart in the superior direction, the differences in all directions between the shifted and original plans were statistically meaningful. When the isocenter shifted to the left or anterior direction, the doses to the OARs decreased. In contrast, shifts to the right, inferior, or posterior directions led to increased doses.

**TABLE 2:** Mean ± standard deviation of dosimetric parameters for the target and OARs in the new plans generated after isocenter shifts of 2.5 mm.

		Direction						
Structure	Parameters	Origin	*x* − 2.5	*x* + 2.5	*y* − 2.5	*y* + 2.5	*z* − 2.5	*z* + 2.5
PTV	$V_{95}$(%)	95.5 ± 1.23	96.09 ± 1.23	93.79 ± 1.37	95.82 ± 1.18	94.26 ± 1.31	96.24 ± 1.11	93.23 ± 1.42
	$D_{98}$(cGy)	4393.23 ± 216.43	4490.97 ± 201.13	4141.83 ± 243.27	4459.33 ± 196.08	4220.9 ± 239.21	4516.83 ± 170.81	4077.87 ± 271.43
	$D_{95}$(cGy)	4780.35 ± 97.24	4802.91 ± 127.8	4610.34 ± 182.14	4782.32 ± 132.14	4652.8 ± 172.95	4815.24 ± 120.66	4563.75 ± 178.44
	$D_{2}$(cGy)	5422.41 ± 45.35	**5428.6** ± **57.9**	5439.51 ± 44.6	5428.58 ± 48.32	5425.8 ± 44.72	5436.19 ± 64.33	5433.73 ± 42.94
Left lung	$V_{5}$(%)	50.45 ± 3.88	52.57 ± 3.79	48.49 ± 3.92	51.6 ± 3.87	49.27 ± 3.92	51.71 ± 3.78	49.12 ± 4.04
	$V_{20}$(%)	19.33 ± 3.37	21.54 ± 3.43	17.31 ± 3.36	21.09 ± 3.29	17.63 ± 3.41	21.67 ± 4.41	16.95 ± 3.33
	$D_{mean}$(cGy)	1098.22 ± 313.21	1191.47 ± 142.36	1016.52 ± 122.19	1164.61 ± 133.51	1035.89 ± 128.6	1192.23 ± 139.42	1007.04 ± 122.34
Right breast	$V_{10}$(%)	6.49 ± 4.19	7.44 ± 4.43	5.63 ± 3.94	6.86 ± 4.32	6.16 ± 4.12	7.3 ± 4.53	5.98 ± 4.02
	$D_{mean}$(cGy)	454.72 ± 110.21	472.62 ± 114.21	439.02 ± 106.25	461.4 ± 111.4	449.47 ± 109.71	471.59 ± 115.67	443.69 ± 107.71
Heart	$V_{5}$(%)	29.49 ± 10.51	30.72 ± 10.5	28.41 ± 10.46	30 ± 10.79	**29.29** ± **10.22**	32.6 ± 10.52	26.54 ± 10.36
	$V_{10}$(%)	12.06 ± 5.01	13.17 ± 5.18	11.06 ± 4.81	12.33 ± 5.24	**11.97** ± **4.84**	14.42 ± 5.33	9.99 ± 4.6
	$V_{20}$(%)	5.07 ± 2.73	5.92 ± 2.99	4.32 ± 2.46	5.2 ± 2.82	4.98 ± 2.69	6.64 ± 3.14	3.77 ± 2.32
	$D_{mean}$(cGy)	569.96 ± 122.27	601.18 ± 128.43	543.03 ± 115.17	578.32 ± 127.17	565.32 ± 118.75	633.08 ± 134.38	517.33 ± 110.41

The values in bold indicate not statistically significant between this dose parameter and the original dose parameter.

**TABLE 3:** Mean ± standard deviation of dosimetric parameters for the target and OARs in the new plans generated after isocenter shifts of 5 mm.

		Direction						
Structure	Parameters	Origin	*x *− 5	*x* + 5	*y*− 5	*y* + 5	*z*− 5	*z* + 5
PTV	$V_{95}$(%)	95.5 ± 1.23	**95.34** ± **1.13**	90.92 ± 1.8	94.9 ± 1.22	92.25 ± 1.72	95.01 ± 1.25	89.37 ± 2.11
	$D_{98}$(cGy)	4393.23 ± 216.43	4441.94 ± 170.43	3761.67 ± 284.78	**4363.22** ± **193.04**	3932.54 ± 293.35	**4411.73** ± **183.81**	3636.05 ± 337.85
	$D_{95}$(cGy)	4780.35 ± 97.24	**4767.02** ± **80.43**	4389.65 ± 168.14	4735.78 ± 88.97	4499.75 ± 159.73	4740.01 ± 90.9	4282.69 ± 206.36
	$D_{2}$(cGy)	5422.41 ± 45.35	**5433.68** ± **65.87**	5463.43 ± 45.07	5438.58 ± 50.33	5432.59 ± 43.77	5451.41 ± 85.41	5455.8 ± 44.04
Left Lung	$V_{5}$(%)	50.45 ± 3.88	54.46 ± 3.88	46.73 ± 4	52.59 ± 4	48.12 ± 3.97	52.87 ± 3.81	47.73 ± 4.23
	$V_{20}$(%)	19.33 ± 3.37	23.32 ± 3.66	15.5 ± 3.37	22.67 ± 3.57	16.13 ± 3.4	23.77 ± 3.68	14.57 ± 3.25
	$D_{mean}$(cGy)	1098.22 ± 313.21	1279.95 ± 131.7	947.32 ± 114	1227.82 ± 147.67	982.87 ± 124.16	1282.21 ± 156.52	923.16 ± 112.83
Right Breast	$V_{10}$(%)	6.49 ± 4.19	8.34 ± 4.66	4.86 ± 3.67	7.34 ± 4.46	5.94 ± 4.04	8.15 ± 4.86	5.7 ± 4.06
	$D_{mean}$(cGy)	454.72 ± 110.21	489.6 ± 101.52	424.77 ± 101.52	464.94 ± 111.61	445.92 ± 108.82	490.22 ± 122	436.87 ± 108.35
Heart	$V_{5}$(%)	29.49 ± 10.51	31.78 ± 10.52	27.4 ± 10.43	30.68 ± 11.03	**29.44** ± **9.98**	35.52 ± 10.67	23.72 ± 10.13
	$V_{10}$(%)	12.06 ± 5.01	14.18 ± 5.38	10.16 ± 4.67	12.76 ± 5.41	**12.12** ± **4.76**	16.83 ± 5.82	8.17 ± 4.17
	$V_{20}$(%)	5.07 ± 2.73	6.73 ± 3.23	3.64 ± 2.22	5.38 ± 2.88	**4.97** ± **2.68**	8.36 ± 3.67	2.76 ± 1.87
	$D_{mean}$(cGy)	569.96 ± 122.27	630.22 ± 135.98	519.56 ± 110.05	591.11 ± 136.27	**565.67** ± **116.32**	703.02 ± 151.99	472.2 ± 99.67

The values in bold indicate not statistically significant between this dose parameter and the original dose parameter.

### 3.2 Effect of setup errors on dosimetry with different breast size

Tables 4 and 5 summarize the mean ± standard deviation of absolute changes in dosimetric parameters for the target and OARs in the large and small breast groups compared to the original plan, following isocenter shifts of 2.5 mm or 5 mm. These tables also highlight the statistical significance of the differences between the two groups. For the target, when a 2.5 mm isocenter shift was introduced, the dosimetric variation for $\Delta D_{2}$ in the inferior and posterior direction was significantly greater in the small breast group compared to the large breast group. However, no statistically meaningful differences were observed in the dosimetric variations of other parameters. When isocenter errors increased to 5 mm, the overall dosimetric variations were greater in the small breast group compared to the large breast group. Moreover, notable statistical differences were observed between the two groups for $\Delta V_{95}$ in the left, anterior, and posterior shifts, $\Delta D_{95}$ in the posterior shifts, and $\Delta D_{2}$ in the inferior shifts. The treatment plans for patients with large breasts demonstrated greater robustness in maintaining PTV dose consistency under the same isocenter errors. Robustness, in this context, refers to the sensitivity of the planned dose to uncertainties, such as patient positioning variations, with fewer dose changes indicating higher robustness.^30^

**TABLE 4:** Mean ± standard deviation of the changes in dosimetric parameters for the target and OARs in the large breast and small breast groups after isocenter shifts of 2.5 mm.

			Direction					
Structure	Parameters	Breast Size	*x *− 2.5	*x *+ 2.5	*y *− 2.5	*y* + 2.5	*z *− 2.5	*z* + 2.5
PTV	$V_{95}$(%)	Large	0.67 ± 0.24	1.67 ± 0.23	0.38 ± 0.24	1.25 ± 0.27	0.87 ± 0.31	2.02 ± 0.47
		Small	0.69 ± 0.43	1.74 ± 0.62	0.29 ± 0.2	1.26 ± 0.48	0.64 ± 0.46	2.52 ± 0.76
		*p*‐Value	0.871	0.673	0.274	0.879	0.129	0.056
	$D_{98}$(cGy)	Large	114.74 ± 51.64	248.47 ± 68.19	79.25 ± 41.23	186.77 ± 48.14	153.11 ± 87.35	300.4 ± 103.26
		Small	106.83 ± 55.65	234.33 ± 89.49	53.72 ± 38.68	158.83 ± 60.9	100.56 ± 77.88	330.31 ± 109.86
		*p*‐Value	0.693	0.566	0.094	0.219	0.084	0.426
	$D_{95}$(cGy)	Large	47.38 ± 28.54	146.75 ± 44.06	29.17 ± 19.93	105.2 ± 27.19	63.31 ± 37.75	180.51 ± 70.39
		Small	46.51 ± 36.33	135.62 ± 49.57	19.28 ± 15.28	94.77 ± 40.29	44.64 ± 32.74	198.56 ± 66.57
		*p*‐Value	0.913	0.679	0.286	0.421	0.18	0.48
	$D_{2}$(cGy)	Large	10.48 ± 4.37	18.7 ± 5.98	3.99 ± 3.14	4.53 ± 3.01	11.36 ± 7.16	15.91 ± 7.55
		Small	23.62 ± 32.25	15.57 ± 8.49	9.51 ± 7.87	6.51 ± 5.76	34.03 ± 32.15	15.85 ± 13.78
		*p*‐Value	0.248	0.171	$0.006^{*}$	0.218	$0.015^{*}$	0.988
Left Lung	$V_{5}$(%)	Large	1.93 ± 0.42	1.87 ± 0.45	1.19 ± 0.23	1.23 ± 0.29	1.11 ± 0.41	1.14 ± 0.4
		Small	2.31 ± 0.67	2.03 ± 0.5	1.12 ± 0.33	1.13 ± 0.31	1.41 ± 0.21	1.5 ± 0.37
		*p*‐Value	0.094	0.388	0.328	0.296	$0.027^{*}$	$0.033^{*}$
	$V_{20}$(%)	Large	2.13 ± 0.31	2.02 ± 0.27	1.94 ± 0.28	1.86 ± 0.27	2.28 ± 0.37	2.32 ± 0.36
		Small	2.3 ± 0.76	2.02 ± 0.35	1.58 ± 0.34	1.53 ± 0.21	2.4 ± 0.27	2.44 ± 0.31
		*p*‐Value	0.472	0.954	$0.006^{*}$	$0.012^{*}$	0.349	0.323
	$D_{mean}$(cGy)	Large	87.12 ± 10.78	79.45 ± 13.11	71.87 ± 10.96	66.95 ± 10.51	89.25 ± 16.55	86.31 ± 18.16
		Small	99.38 ± 36.04	83.94 ± 18.34	60.91 ± 12.74	57.71 ± 13.1	98.79 ± 12.1	96.06 ± 12.02
		*p*‐Value	0.248	0.381	$0.025^{*}$	$0.048^{*}$	0.112	0.115
Right Breast	$V_{10}$(%)	Large	1 ± 0.26	0.92 ± 0.24	0.44 ± 0.3	0.35 ± 0.26	0.82 ± 0.56	0.72 ± 0.41
		Small	0.9 ± 0.42	0.8 ± 0.44	0.43 ± 0.29	0.37 ± 0.26	1.04 ± 0.9	0.86 ± 0.86
		*p*‐Value	0.38	0.275	0.939	0.711	0.557	0.913
	$D_{mean}$(cGy)	Large	18.06 ± 6.46	16.45 ± 4.79	6.78 ± 5.14	5.27 ± 4.62	14.81 ± 12.91	11.63 ± 8.09
		Small	17.74 ± 6.02	14.94 ± 5.3	6.92 ± 6.07	5.84 ± 5.33	20.38 ± 19.19	15.43 ± 18.01
		*p*‐Value	0.913	0.372	0.983	0.948	0.528	0.913
Heart	$V_{5}$(%)	Large	1.37 ± 0.33	1.27 ± 0.29	0.71 ± 0.35	0.48 ± 0.29	3.14 ± 0.45	3 ± 0.48
		Small	1.11 ± 0.45	0.88 ± 0.35	0.67 ± 0.43	0.62 ± 0.49	3.09 ± 0.46	2.88 ± 0.4
		*p*‐Value	0.063	$0.006^{*}$	0.783	0.514	0.694	0.393
	$V_{10}$(%)	Large	1.2 ± 0.2	1.14 ± 0.19	0.43 ± 0.35	0.3 ± 0.25	2.48 ± 0.56	2.22 ± 0.55
		Small	1.02 ± 0.47	0.887 ± 0.31	0.35 ± 0.24	0.38 ± 0.25	2.24 ± 0.51	1.91 ± 0.48
		*p*‐Value	0.122	$0.012^{*}$	0.472	0.356	0.089	$0.027^{*}$
	$V_{20}$(%)	Large	0.96 ± 0.2	0.9 ± 0.18	0.21 ± 0.17	0.18 ± 0.14	1.77 ± 0.46	1.5 ± 0.43
		Small	0.75 ± 0.38	0.6 ± 0.32	0.14 ± 0.09	0.13 ± 0.08	1.38 ± 0.48	1.09 ± 0.48
		*p*‐Value	$0.031^{*}$	$0.003^{*}$	0.171	0.2	$0.005^{*}$	$0.004^{*}$
	$D_{mean}$(cGy)	Large	34.47 ± 7.08	31.48 ± 6.95	11.58 ± 6.84	8.62 ± 7.11	67.74 ± 14.03	57.65 ± 13.31
		Small	27.96 ± 14.76	22.4 ± 10.03	8.27 ± 5.52	6.86 ± 4.67	58.49 ± 16.34	47.63 ± 13.59
		*p*‐Value	0.094	$0.008^{*}$	0.327	0.379	0.053	$0.023^{*}$

The values in bold indicate and marked with an asterisk (*) statistically significant between large breast and small breast groups.

**TABLE 5:** Mean  ±  standard deviation of the changes in dosimetric parameters for the target and OARs in the large breast and small breast groups after isocenter shifts of 5 mm.

			Direction					
Structure	Parameters	Breast Size	*x* − 5	*x* + 5	*y* − 5	*y* + 5	*z* − 5	*z* + 5
PTV	$V_{95}$(%)	Large	0.41 ± 0.26	4.14 ± 0.51	0.66 ± 1.01	2.99 ± 0.84	0.46 ± 0.39	5.27 ± 1.35
		Small	0.65 ± 0.48	5.02 ± 1.5	0.65 ± 0.34	3.5 ± 1.11	1.32 ± 0.94	6.97 ± 1.69
		*p*‐Value	0.072	$0.037^{*}$	0.306	0.157	$0.002^{*}$	$0.009^{*}$
	$D_{98}$(cGy)	Large	115.17 ± 84.76	503.97 ± 147.09	105.08 ± 159.85	451.65 ± 147.41	144.92 ± 131.75	690.4 ± 194.91
		Small	88.95 ± 64.48	659.14 ± 198.33	67.09 ± 46.95	469.75 ± 161.82	145.49 ± 108.06	823.94 ± 203.44
		*p*‐Value	0.306	0.33	0.828	0.586	0.647	0.083
	$D_{95}$(cGy)	Large	29.19 ± 26.41	379.59 ± 101.65	53.8 ± 75.68	278.57 ± 95.08	36.12 ± 38.98	458.89 ± 163.85
		Small	45.59 ± 31.48	401.82 ± 121.06	47.53 ± 25.24	282.63 ± 97.94	93.04 ± 73.69	536.44 ± 148.46
		*p*‐Value	0.078	0.589	0.5	0.913	$0.002^{*}$	0.196
	$D_{2}$(cGy)	Large	18.39 ± 7.08	42.08 ± 7.94	10.54 ± 8.14	11.16 ± 6.82	25.33 ± 16.55	36.52 ± 17.35
		Small	37.35 ± 38.66	39.97 ± 16.24	21.86 ± 14.5	11.57 ± 11.49	61.57 ± 62.02	31.39 ± 30.47
		*p*‐Value	0.267	0.433	$0.009^{*}$	0.472	0.058	0.349
Left Lung	$V_{5}$(%)	Large	0.37 ± 0.87	3.51 ± 1.12	2.36 ± 0.45	2.35 ± 0.8	2.25 ± 0.75	2.34 ± 0.9
		Small	4.33 ± 0.83	3.92 ± 0.89	2.21 ± 0.61	2.3 ± 0.69	2.77 ± 0.5	3.08 ± 0.67
		*p*‐Value	$0.049^{*}$	0.313	0.339	0.837	0.058	$0.033^{*}$
	$V_{20}$(%)	Large	4.1 ± 0.88	3.77 ± 0.91	3.87 ± 0.56	3.41 ± 1	4.48 ± 0.7	4.63 ± 0.81
		Small	4.3 ± 0.68	3.96 ± 0.71	3.2 ± 0.69	2.99 ± 0.61	4.71 ± 0.77	4.87 ± 0.61
		*p*‐Value	0.378	0.679	$0.006^{*}$	0.085	0.357	0.373
	$D_{mean}$(cGy)	Large	174.74 ± 37.15	144.72 ± 39.09	144.44 ± 20.15	120.06 ± 35.72	181.65 ± 33.71	164.92 ± 37.56
		Small	188.71 ± 35.6	158.77 ± 36.66	127.18 ± 26.76	110.67 ± 24.43	196.46 ± 33.11	185.2 ± 23.12
		*p*‐Value	0.184	0.372	0.094	0.231	0.17	0.103
Right Breast	$V_{10}$(%)	Large	1.9 ± 0.54	1.74 ± 0.62	0.88 ± 0.52	0.57 ± 0.5	1.65 ± 1.2	1.38 ± 0.76
		Small	1.79 ± 0.84	1.52 ± 0.89	0.96 ± 0.59	0.65 ± 0.48	2.03 ± 1.76	1.57 ± 1.63
		*p*‐Value	0.606	0.319	0.731	0.472	0.679	0.67
	$D_{mean}$(cGy)	Large	35.46 ± 13.39	30.9 ± 11.59	18.52 ± 21.85	8.58 ± 8.52	32.65 ± 27.16	21.26 ± 13.33
		Small	34.32 ± 13.05	29 ± 10.47	15.33 ± 12.6	10.3 ± 10	40.65 ± 38.54	26.62 ± 32.78
		*p*‐Value	0.913	0.616	0.711	0.679	0.744	0.811
Heart	$V_{5}$(%)	Large	2.57 ± 0.69	2.41 ± 0.48	1.57 ± 0.72	0.77 ± 0.45	6.28 ± 0.86	5.94 ± 0.98
		Small	2.03 ± 0.67	1.76 ± 0.68	1.47 ± 0.97	1.19 ± 0.87	5.85 ± 1.5	5.59 ± 0.89
		*p*‐Value	$0.033^{*}$	$0.01^{*}$	0.772	0.095	0.42	0.225
	$V_{10}$(%)	Large	2.34 ± 0.48	4.49 ± 0.7	3.04 ± 0.94	1.33 ± 1.08	5.22 ± 1.42	9.34 ± 2.12
		Small	1.91 ± 0.65	1.66 ± 0.61	0.81 ± 0.63	0.71 ± 0.45	4.45 ± 1.38	3.56 ± 0.96
		*p*‐Value	$0.048^{*}$	$0.001^{*}$	$0.001^{*}$	0.151	0.112	$0.001^{*}$
	$V_{20}$(%)	Large	1.89 ± 0.45	1.69 ± 0.39	0.48 ± 0.38	0.27 ± 0.25	3.72 ± 0.95	2.78 ± 0.8
		Small	1.44 ± 0.64	1.17 ± 0.6	0.3 ± 0.25	0.24 ± 0.13	2.88 ± 1.114	2.03 ± 0.88
		*p*‐Value	$0.028^{*}$	$0.008^{*}$	0.139	0.728	$0.012^{*}$	$0.006^{*}$
	$D_{mean}$(cGy)	Large	67.28 ± 15.71	125.69 ± 25.68	85.89 ± 42.53	40.78 ± 43.34	153.22 ± 39.57	252.71 ± 54.75
		Small	53.23 ± 24.2	42.4 ± 19.98	18.95 ± 11.48	12.63 ± 8.34	121.75 ± 41.99	88.24 ± 26.27
		*p*‐Value	0.071	$0.001^{*}$	$0.001^{*}$	$0.012^{*}$	$0.018^{*}$	$0.001^{*}$

The values in bold and marked with an asterisk (*) indicate statistically significant between large breast and small breast groups.

**FIGURE 1:**
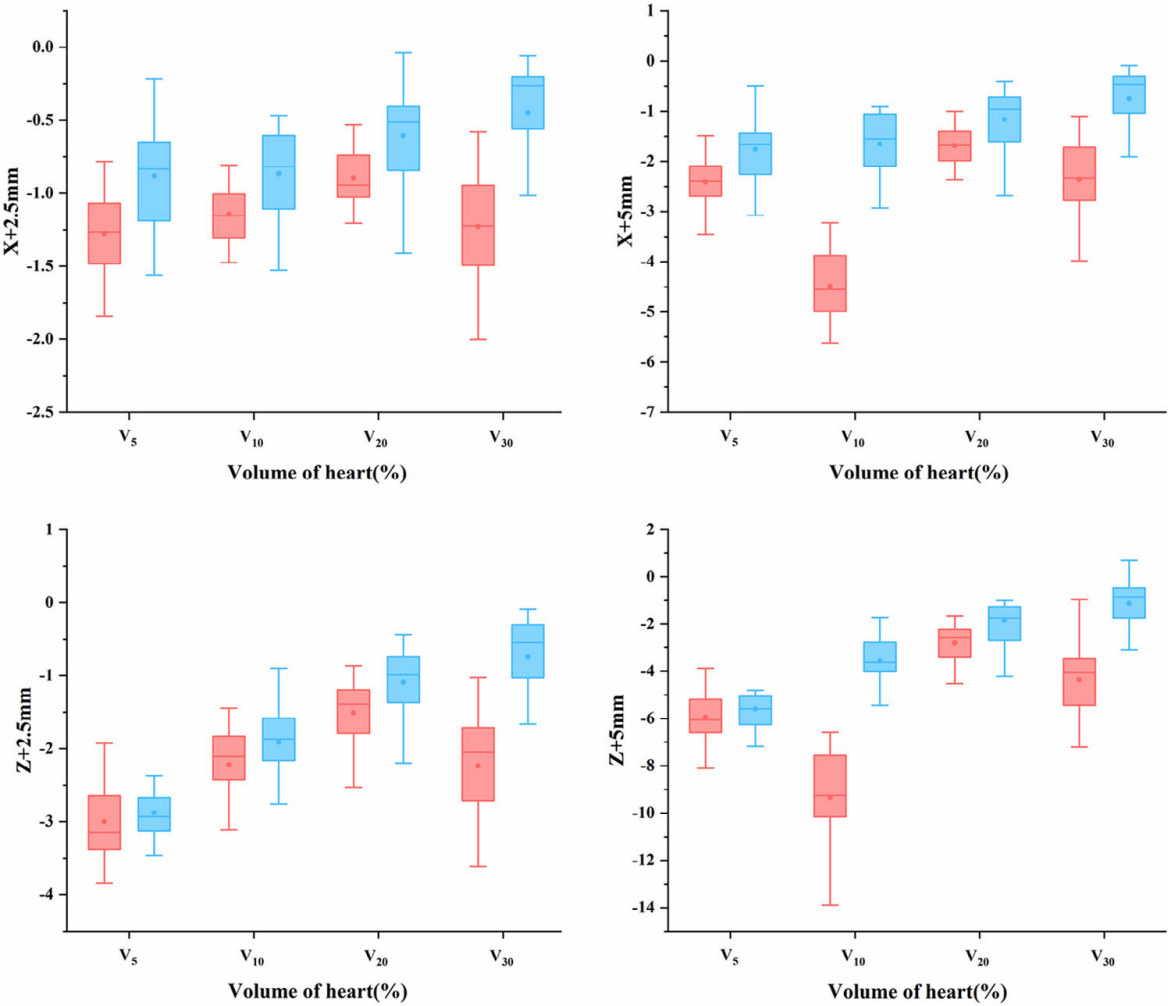
Statistical comparisons between large breast (red) and small breast (blue) group of heart dose variations when the setup error against the left (*x*) and anterior (*z*) directions.

For the heart, when an isocenter error of 2.5 mm was introduced, the dosimetric variation in the small breast group was smaller than that in the large breast group. Statistically variations between the groups were identified for $V_{5}$ in left shifts, $V_{10}$ in left and anterior shifts, $V_{20}$ in left, right, anterior, and posterior shifts, and $\Delta D_{mean}$ in left and anterior shifts. As the isocenter error increased, the differences between the two groups became more evident. To provide a clearer depiction of the differences in heart dose robustness, boxplots of heart dose variations for both groups under left and anterior isocenter shifts are presented in Figure 1. For the left lung, dosimetric variations in the small breast group were more obvious than those in the large breast group for left‐right and anterior‐posterior shifts. Specifically, statistical differences in $V_{5}$ were detected between the groups for anterior‐posterior shifts with a 2.5 mm isocenter error and for right and anterior shifts with a 5 mm isocenter error. However, for superior‐inferior shifts, the dosimetric variation in the small breast group was less than that in the large breast group. Notable differences in $V_{20}$ were noted for superior‐inferior shifts with a 2.5 mm isocenter error and for inferior shifts with a 5 mm isocenter error.

### 3.3 Effect of setup errors on NTCP of OARs

The EUD and NTCP values for the heart and left lung under 2.5 mm and 5 mm setup errors are summarized in Tables 6 and 7. Regardless of breast size, the NTCP for the heart and left lung increased when the isocenter shifted to the right, inferior, or posterior directions, and decreased with shifts in the opposite directions. With the exception of the EUD and NTCP values for the heart in the small breast group under a 5 mm superior shift, all other directions and shift magnitudes demonstrated statistically significant differences in heart and left lung parameters between the shifted and original plans.

**TABLE 6:** Mean ± standard deviation of radiobiological parameters for the OARs after isocenter shifts of 2.5 mm.

			Direction						
Structure	Breast Size	Parameters	Origin	*x* − 2.5	*x* + 2.5	*y* − 2.5	*y* + 2.5	*z* − 2.5	*Z* + 2.5
Heart	Large	Eud (Gy)	10.34 ± 1.67	11.22 ± 1.69	9.49 ± 1.62	10.59 ± 1.71	10.13 ± 1.64	11.96 ± 1.71	8.9 ± 1.62
		NTCP	2.05E‐6 ± 3.67E‐6	5E‐6 ± 8.87E‐6	8.23E‐7 ± 4.66E‐6	2.7E‐6 ± 4.66E‐6	1.66E‐6 ± 3.14E‐6	9.41E‐6 ± 1.48E‐5	4.29E‐7 ± 8.08E‐7
	Small	Eud(Gy)	8.49 ± 2.14	9.38 ± 2.31	7.76 ± 1.2	8.64 ± 2.19	8.41 ± 2.12	10.16 ± 2.27	7.08 ± 1.98
		NTCP	7.16E‐7 ± 1.9E‐6	2.16E‐6 ± 5.73E‐6	2.46E‐7 ± 6.43E‐7	9.41E‐7 ± 2.59E‐6	6.46E‐7 ± 1.71E‐6	4.85E‐6 ± 1.36E‐5	1.09E‐7 ± 2.89E‐7
Left Lung	Large	Eud(Gy)	8.78 ± 1.41	9.63 ± 1.5	7.99 ± 1.32	9.47 ± 1.47	8.12 ± 1.36	9.67 ± 1.56	7.91 ± 1.29
		NTCP	0.049 ± 0.059	0.1 ± 0.116	0.024 ± 0.029	0.086 ± 0.098	0.028 ± 0.036	0.106 ± 0.125	0.0217 ± 0.027
	Small	Eud(Gy)	8.9 ± 1.22	9.89 ± 1.43	8.09 ± 1.08	9.51 ± 1.26	8.34 ± 1.18	9.9 ± 1.3	7.95 ± 1.12
		NTCP	0.046 ± 0.04	0.113 ± 0.11	0.021 ± 0.017	0.076 ± 0.024	0.028 ± 0.024	0.104 ± 0.089	0.019 ± 0.016

**TABLE 7:** Mean ± standard deviation of radiobiological parameters for the OARs after isocenter shifts of 5 mm.

			Direction						
Structure	Breast Size	Parameters	Origin	*x* − 5	*x* + 5	*y* − 5	*y* + 5	*z* − 5	*z* + 5
Heart	Large	Eud(Gy)	10.34 ± 1.67	12.13 ± 1.76	8.74 ± 1.61	10.86 ± 1.77	10.06 ± 1.63	13.72 ± 1.84	7.65 ± 1.56
		NTCP	2.05E‐6 ± 3.67E‐6	1.2E‐5 ± 2.15E‐5	3.45E‐7 ± 6.5E‐7	3.72E‐6 ± 6.23E‐6	1.56E‐6 ± 3.08E‐6	4.47E‐5 ± 6.53E‐5	9.8E‐8 ± 2.08E‐7
	Small	Eud(Gy)	8.49 ± 2.14	10.15 ± 2.47	7.09 ± 1.87	8.85 ± 2.28	**8.39** ± **2.14**	11.81 ± 2.75	5.88 ± 1.82
		NTCP	7.16E‐7 ± 1.9E‐6	6.43E‐6 ± 1.79E‐5	8.6E‐8 ± 2.24E‐7	1.34E‐6 ± 3.75E‐6	**6.54E‐7** ± **1.74E‐6**	2.77E‐5 ± 7.21E‐5	1.61E‐8 ± 3.96E‐8
Left Lung	Large	Eud(Gy)	8.78 ± 1.41	10.52 ± 1.66	7.41 ± 1.22	10.12 ± 1.69	7.63 ± 1.28	10.63 ± 1.65	7.16 ± 1.15
		NTCP	0.049 ± 0.059	0.2 ± 0.23	0.01 ± 0.015	0.15 ± 0.16	0.017 ± 0.022	0.22 ± 0.25	0.009 ± 0.012
	Small	Eud(Gy)	8.9 ± 1.22	10.77 ± 1.49	7.36 ± 0.98	10.17 ± 1.29	7.84 ± 1.15	10.81 ± 1.58	7.08 ± 1.02
		NTCP	0.046 ± 0.04	0.22 ± 0.2	0.009 ± 0.007	0.13 ± 0.11	0.02 ± 0.015	0.22 ± 0.19	0.007 ± 0.006

The values in bold indicate not statistically significant between this radiobiological parameter and the original radiobiological parameter.

When comparing NTCP deviations between the two groups, the large breast group exhibited greater variations in the NTCP of the heart compared to the small breast group. These differences were statistically evident for 2.5 mm and 5 mm isocenter shifts in the superior‐inferior direction ($p_{2.5,superior}=0.018$, $p_{2.5,inferior}=0.011$, $p_{5,superior}=0.028$, $p_{5,inferior}=0.018$). For the ipsilateral lung, however, differences in NTCP changes between the two groups were not statistically significantly.

### 3.4 Actual setup errors in the clinic

**FIGURE 2:**
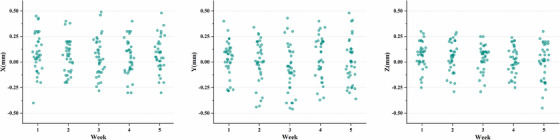
The statistics of setup errors in different directions (*x*, *y*, *z*) in clinical treatment.

Figure 2 illustrates the setup errors observed in 36 patients over 180 CBCT scans during clinical treatment. The mean absolute setup errors in the x, y, and z directions were calculated as 1.47 ± 1.12 mm, 1.64 ± 1.23 mm, and 1.19 ± 0.86 mm, respectively, all within the clinically acceptable threshold of less than 5 mm. In the large breast group, the mean absolute setup errors were determined to be 1.57 ± 1.17 mm, 1.64 ± 1.17 mm, and 1.26 ± 0.88 mm for the x, y, and z directions, respectively. In the small breast group, the corresponding values were measured as 1.38 ± 1.07 mm, 1.64 ± 1.30 mm, and 1.11 ± 0.84 mm. The differences between the two groups did not reach statistical significance, suggesting that breast size does not have a notable impact on the occurrence of setup errors. Table 8 presents the mean absolute values and standard deviations of dosimetric parameters for the target and organs at risk across five clinical setup errors. Compared to the original plan, PTV coverage was significantly lower, while heart dose was generally higher.

**TABLE 8:** Mean ± standard deviation of dosimetric parameters for the target and OARs under clinical setup errors.

		Direction		
Structure	Parameters	Origin	Average	*p*‐Value
PTV	$V_{95}$(%)	95.5 ± 1.23	94.48 ± 2.4	**0**
	$D_{98}$(cGy)	4393.23 ± 216.43	4262.19 ± 340.09	**0.001**
	$D_{95}$(cGy)	4780.35 ± 97.24	4679.23 ± 240.76	**0**
	$D_{2}$(cGy)	5422.41 ± 45.35	5438.26 ± 54.27	**0**
Left lung	$V_{5}$(%)	50.45 ± 3.88	50.14 ± 4.29	0.291
	$V_{20}$(%)	19.33 ± 3.37	19.2 ± 4.57	0.738
	$D_{\mathrm{mean}}$(cGy)	1098.22 ± 313.21	1100.3 ± 178.71	0.889
Right breast	$V_{10}$(%)	6.49 ± 4.19	6.47 ± 4.34	0.875
	$D_{\mathrm{mean}}$(cGy)	454.72 ± 110.21	454.92 ± 111.78	0.838
Heart	$V_{5}$(%)	29.49 ± 10.51	29.91 ± 10.22	0.167
	$V_{10}$(%)	12.06 ± 5.01	12.3 ± 5.1	0.239
	$V_{20}$(%)	5.07 ± 2.73	5.17 ± 2.86	0.505
	$D_{\mathrm{mean}}$(cGy)	569.96 ± 122.27	578.69 ± 125.62	0.161

The values in bold indicate statistically significant between the mean dose parameter of the five plans and the original dose parameter.

## 4 DISCUSSION

This study investigated the effects of setup errors on the dosimetry and radiobiological parameters in left‐sided BC patients with varying breast sizes undergoing conventionally fractionated VMAT radiotherapy. Simulated setup errors were introduced through isocenter shifts of 2.5 mm and 5 mm along six directions on the x, y, and z axes. The results showed that the small breast group provided superior heart protection, whereas the large breast group demonstrated greater robustness in PTV coverage under 5 mm isocenter errors. These findings were consistent across both dosimetric and radiobiological analyses. However, the influence of breast volume on the ipsilateral lung under setup errors warrants further investigation, particularly regarding the direction of the isocenter shifts.

In our study, with a 2.5 mm setup error, breast size had a relatively small impact on PTV dose coverage. However, when the setup error increased to 5 mm, breast size markedly influenced PTV dose coverage in the x and z directions. We attribute this to the steep dose gradient required between the target and OARs to ensure precise dose coverage of the target while minimizing exposure to the OARs. In the presence of setup errors, such dose gradients result in rapid dose variations. The smaller PTV in the small breast group leads to a greater proportion of overlap with OARs, which substantially affects dose accuracy. Conversely, in the large breast group, the heart dose is more significantly impacted. Previous studies have shown that treatment plans for patients with larger breasts involve more subfields, resulting in a larger intensity‐modulated radiation field and increased scatter radiation, which negatively impacts OARs.^31^

To further investigate the risks of radiotherapy to OARs, we evaluated radiobiological parameters. The Niemierko model was applied to calculate the Dose‐Volume Histogram (DVH) and derive the NTCP values for the heart and left lung. Consistent with the dosimetric findings, the same setup errors had a more pronounced impact on the NTCP of the heart in patients with larger breasts. However, the changes in biological effects did not exhibit a linear relationship with the dosimetric deviations. Under 2.5 mm and 5 mm setup errors, the median ΔNTCP for the heart in the large‐breast group was 27.7 times and 19.3 times higher than that in the small‐breast group, respectively. In contrast, the median differences in Dmean were only 1.3 times and 2.8 times. Similarly, with clinically observed setup errors, the ΔNTCP was 11 times higher, compared to a 1.3‐fold difference in Dmean. This suggests that relying solely on dosimetric parameters may underestimate the risk of cardiac toxicity. In addition, the left lung showed higher dosimetric parameters and NTCP values than the heart. This is primarily due to the dynamic intensity modulation of VMAT. While VMAT improves target dose conformality, it increases the low‐dose irradiation volume in the ipsilateral lung. Therefore, incorporating radiobiological factors is essential when managing setup errors in clinical practice.

Image‐Guided Radiation Therapy (IGRT) is widely utilized to precisely localize the target and correct patient setup errors before delivering clinical radiotherapy. With the incorporation of imaging guidance, average setup errors are typically reduced to 1–5 mm.^32^, ^33^ however, completely eliminating such errors remains challenging. Costin et al. reviewed various image‐guidance techniques employed over the past three decades for breast cancer radiotherapy. Despite advancements, many studies have reported mean setup errors exceeding 2.5 mm.^10^ According to Batumalai et al.’s analysis of literature on setup errors in breast radiotherapy using CBCT, the range of setup errors was 1.2–5.7 mm in the x‐direction, 1.3–3.8 mm in the y‐direction, and 0.7–5.7 mm in the z‐direction.^32^ In this study, setup errors exceeding 2.5 mm in at least one direction were observed in 67 instances, representing 44.67% of all cases. Investigating the impact of setup errors greater than 2.5 mm or as large as 5 mm on breast cancer patients with varying breast sizes is therefore of critical importance.

Radiation‐induced heart damage is a significant factor limiting the long‐term survival of breast cancer patients, increasing cardiovascular mortality by 24% to 62%. This risk escalates with higher radiation doses.^34^, ^35^, ^36^, ^37^ Darby et al. conducted a statistical analysis of 2,168 patients and found that for every 1 Gy increase in mean heart dose (MHD), the incidence of coronary events rose linearly by 7.4%.^36^ Similarly, Van den Bogaard et al. conducted a longitudinal study tracking 910 female breast cancer patients who underwent radiotherapy. Their findings revealed a correlation between the probability of acute coronary events within 9 years and the cardiac radiation dose, yielding results consistent with previous studies.^37^ In our study, setup errors in the right, inferior, and posterior directions led to a greater rise in MHD for patients with larger breasts compared to those with smaller breasts. When the setup error reached 5 mm, the maximum MHD increase was as high as 2.5 Gy, potentially elevating the estimated risk of coronary events by 18.5%. Currently, no safe threshold for heart dose has been established, emphasizing the critical importance of minimizing even small increases in radiation exposure to the heart.

This study has three limitations. First, soft tissue deformation during treatment was not considered. In our study, all plans employed the “Auto Flash” function of the Monaco TPS to compensate for dose inaccuracies caused by respiratory motion or soft tissue deformation. However, a study by Majia et al. on target dose coverage during radiotherapy for left‐sided breast cancer across different planning systems demonstrated that when soft tissue deformation exceeds 8 mm, the “Auto Flash” feature becomes less effective in optimizing target dose coverage.^38^ They improved target dose robustness by combining “Auto Flash” with a bolus, a strategy that could be explored in future work. Second, only translational errors in the x, y, and z directions were considered in this study, while rotational errors along different axes were not examined. Studies have indicated that isocenter rotations exceeding 2° in any direction can lead to significant dose deviations.^15^, ^39^ Third, the data in this study were derived from a single institution and were relatively limited in size. As a result, it remains unclear whether the observed dosimetric differences between large and small breast groups under clinical setup errors are primarily attributable to breast volume or simply to the larger setup deviations observed in the large‐breast group. Increasing the sample size and incorporating multi‐center data would help to further validate and strengthen our conclusions.

## 5 CONCLUSION

This study demonstrates that for left‐sided breast cancer patients undergoing conventionally fractionated radiotherapy VMAT radiotherapy, setup errors exceeding 2.5 mm result in notable alterations in the doses to both the target and OARs, particularly increasing dose exposure to critical OARs when shifts occur in the right, inferior, and posterior directions. When the setup error reaches or exceeds 2.5 mm, the increase in heart dose and NTCP is more pronounced in patients with larger breasts compared to those with smaller breasts. This effect is particularly evident in the right and posterior directions, leading to a significantly higher cardiac risk. Therefore, for left‐sided breast cancer patients with breast volumes exceeding 975 cm^3^, we recommend implementing stricter setup error thresholds and more frequent imaging guidance. For example, reducing the acceptable setup error to 2.5 mm and performing CBCT verification at least twice per week may be warranted.

## AUTHOR CONTRIBUTIONS

**Chao Zheng**: Conceptualization; methodology; data curation; original draft preparation. **Danting Cai**: Data analysis; data curation; investigation. **Qingsong Zhong**: Conceptualization; supervision. **Wen Dou**: Validation; critical review of the manuscript. **Binbin Yuan**: Data extraction; data curation.

## CONFLICT OF INTEREST STATEMENT

The authors declare no conflicts of interest.
